# A Systematic Review of Experimental Paradigms for Exploring Biased Interpretation of Ambiguous Information with Emotional and Neutral Associations

**DOI:** 10.3389/fpsyg.2017.00171

**Published:** 2017-02-09

**Authors:** Daniel E. Schoth, Christina Liossi

**Affiliations:** Pain Research Laboratory, Department of Psychology, University of SouthamptonSouthampton, UK

**Keywords:** interpretation bias, ambiguous information, ambiguity resolution, experimental paradigm, systematic review

## Abstract

Interpretation biases have been extensively explored in a range of populations, including patients with anxiety and depressive disorders where they have been argued to influence the onset and maintenance of such conditions. Other populations in which interpretation biases have been explored include patients with chronic pain, anorexia nervosa, and alcohol dependency among others, although this literature is more limited. In this research, stimuli with threatening/emotional and neutral meanings are presented, with participant responses indicative of ambiguity resolution. A large number of paradigms have been designed and implemented in the exploration of interpretation biases, some varying in minor features only. This article provides a review of experimental paradigms available for exploring interpretation biases, with the aim to stimulate and inform the design of future research exploring cognitive biases across a range of populations. A systematic search of the experimental literature was conducted in Medline, PsychINFO, Web of Science, CINAHL, and Cochrane Library databases. Search terms were *information, stimuli*, and *ambiguous* intersected with the terms *interpretation* and bias^*^. Forty-five paradigms were found, categorized into those using ambiguous words, ambiguous images, and ambiguous scenarios. The key features, strengths and limitations of the paradigms identified are discussed.

## Introduction

An abundance of research shows humans are capable of a wide range of cognitive biases (Pronin, 2007; Hilbert, 2012), and that perception is not simply a passive process but instead is shaped by many factors including individual expectations, beliefs and memories (Allport, 1955). As such, two people subject to the same information, such as a visual scene or a description of event, may interpret that information in different ways. Ambiguity is ubiquitous in everyday life, and can include information such as words and images that are vague and have multiple meanings, along with facial expressions, behaviors, and comments from other people which are open to interpretation. Interpretation is the process of assigning meaning to an ambiguous stimulus or situation (Wisco and Nolen-Hoeksema, 2010). An interpretation bias may be defined as a tendency to interpret ambiguous information in a consistent manner, which is usually threatening or negative (although positive interpretation biases can also exist yet are much less researched; Hirsch et al., 2016). Schema theory states that cognitive structures (i.e., schemas) guide information processing, that information congruent with such schemas is preferentially elaborated and encoded, and predicts cognitive biases across different forms of information processing: “When specific schemas or a constellation of schemas is activated, their content directly influences the content of a person's perceptions, interpretations, associations, and memories at a given time” (Beck et al., 1985, p. 55). An individual encountering ambiguous information is therefore predicted to interpret that information in a manner congruent with their existing schemas.

Interpretation biases have been extensively studied in relation to psychopathology, where they are argued to have a role in the onset and maintenance of disorders such as anxiety and depression (e.g., Beck and Clarke, 1988; Mathews and Mackintosh, 2000; Clark, 2001; Hirsch and Mathews, 2012; Hirsch et al., 2016). Further to anxious and depressed populations, interpretation biases have been explored in numerous other populations, including patients with chronic pain (Schoth and Liossi, 2016; Schoth et al., 2016), anorexia nervosa (Cardi et al., 2015), alcohol-dependent patients (Woud et al., 2014), and individuals high and low in cancer fear (Miles et al., 2009) among others. Consequently a large number of paradigms have been designed and implemented in the exploration of interpretation biases. Overall there is considerable variation between these paradigms, including the stimuli used, the subcomponent of interpretation measured (activation and/or selection of interpretations made), and whether biases are measured directly (e.g., written responses) or indirectly (e.g., response times). Considering this, the purpose of the present article is to provide the first review of experimental paradigms available for exploring biased interpretation of ambiguous information with both threatening/emotional and neutral interpretations. The key features of each paradigm will be described, along with their strengths and limitations. It is believed the knowledge provided in this review will stimulate and inform the design of future research exploring cognitive biases across a range of populations.

## Methods

### Literature search

The review protocol is available on request. Experimental paradigms included in this review were identified via a search of Web of Science (title), Medline, PsychINFO, CINAHL, (title, subject terms), and Cochrane Library databases (title, abstract, keywords). Search terms were *information, stimuli*, and *ambiguous* intersected with the terms *interpretation* and bias^*^. These terms were combined in the following manner: i. *information* AND *interpretation* AND bias^*^; ii. *stimuli* AND *interpretation* AND bias^*^; iii. *ambiguous* AND *interpretation* AND bias^*^. A scan of the reference lists of all obtained articles was also conducted. All searches were made from database inception until 5th August 2016.

### Inclusion criteria

For inclusion in the review, each paradigm was required to meet the following criteria:

1. Presented in a peer-reviewed and published article or report, available in English language until 5th August 2016.
2. Explored interpretation bias for ambiguous information that has both threatening/emotional and neutral interpretations.
3. Explored interpretation bias in adults (≥18 years old) or children (<18 years old).

### Search results

**Figure 1:**
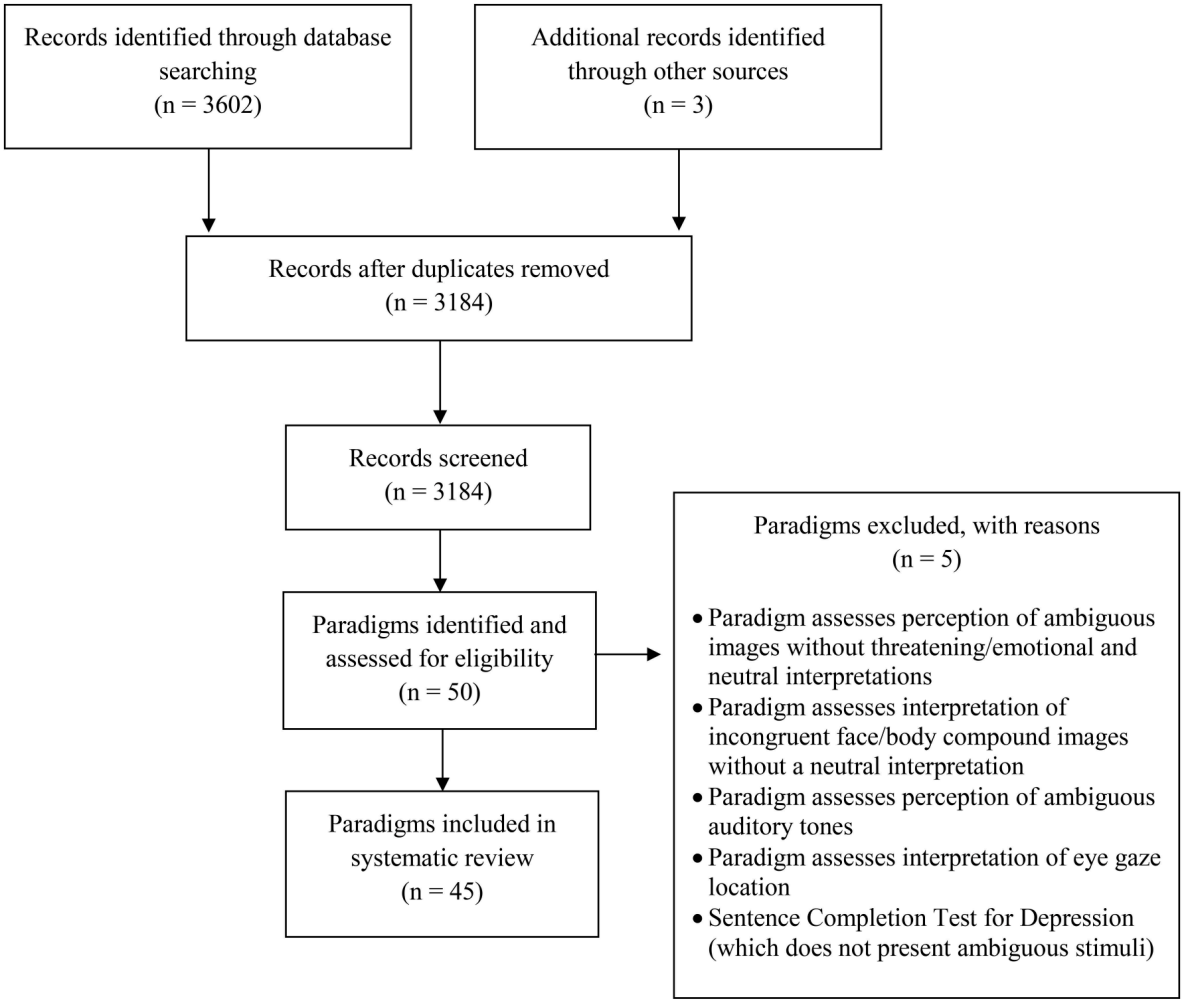
**Flow of records screened and paradigms eligible for inclusion in the systematic review**.

The results of the systematic search identified 3605 records, including 3184 unique records following the removal of duplicates (see Figure 1 for the flow of records screened; Moher et al., 2009). For each record the title and abstract was first read to assess whether the study explored biased interpretation of ambiguous information. If this was the case or if it was unclear, the full text was subsequently read to determine whether the paradigm used met our eligibility criteria. If so, a final check was made to determine whether the paradigm had already been identified as part of our search by consulting our records, and if not was added to the review. This procedure was performed by the first author, and subsequently verified by the second author. A total of 45 eligible paradigms were identified, including 8 using single ambiguous words, 10 using ambiguous images, and 27 using ambiguous scenarios. Table 1 presents a brief description, strengths, and limitations for each paradigm. Reading of full-text articles identified five paradigms which were not eligible for inclusion as they do not allow researchers to explore participants' interpretations of ambiguous information with both threatening/emotional and neutral meanings [i.e., perception of ambiguous images without threatening/emotional and neutral interpretations (e.g., the Necker cube), interpretation of incongruent face/body compound images without a neutral interpretation (i.e., angry and fearful faces and bodies), perception of ambiguous auditory tones, interpretation of eye gaze location, and the Sentence Completion Test for Depression (see Figure 1)]. In the following review, for each paradigm relevant studies using that paradigm are cited. Where examples of experimental research are required for illustration and discussion, the decision was made to include examples from a diverse range of populations. Instead of limiting citations and examples to a single population (e.g., patients with anxiety disorders), this review therefore reflects the breadth of populations within which interpretation biases have been and are currently being explored. It is important to clarify however that the present article provides a review of interpretation bias paradigms, and not a record of every study exploring biased interpretations which is beyond its scope.

**Table 1:** **Characteristics of the interpretation bias paradigms included in the review**.

**Paradigm**	**Brief description [response format]**	**Strengths**	**Limitations**
**SINGLE AMBIGUOUS WORDS**			
Homophone task	Spoken homophones with threatening and neutral associations (e.g., pain/pane) are presented, which participants write down [written response]	Simple to administer without use of a computer	Threatening and neutral associations often have different written and verbal frequencies of use. Often a small number of appropriate stimuli
Homophone task with pictorial responses	Spoken homophones with threatening and neutral associations are presented, followed by pictures corresponding to each interpretation. Participants select the picture best matching the word [verbal response or pointing toward appropriate picture]	Individual differences in reading and writing ability do not affect participant responses. Appropriate for children	Threatening and neutral associations often have different written and verbal frequencies of use. Often a small number of appropriate stimuli
Lexical decision task	Homographs are presented individually as primes, followed by a target word (homophones may also be presented aurally). Participants indicate whether the target is a real-word or not; faster response times to negative compared to neutral target words indicate a priming effect for that meaning [keyboard or response box]	Measure of response times unlikely to be influenced by demand characteristics	Often a small number of appropriate stimuli. Low frequency target words may be subject to guessed responses
Homographic response task - Single word response	Homographs are presented individually, and participants are required to write the first related word they think of [written response]	Simple to administer without use of a computer	Responses may be subject to demand characteristics. Homographic associations vary in their dominance, potentially influencing responses
Homographic response task - Multiple word response	Homographs are presented individually, and participants are required to write multiple associated words [written response]	Simple to administer without use of a computer	Responses may be subject to demand characteristics. Homographic associations vary in their dominance, potentially influencing responses
Sentence generation task	Homographs are presented individually, and participants are required to form a sentence featuring each homograph [written response]	Simple to administer without use of a computer	Responses may be subject to demand characteristics
Word stem completion task	Participants are presented with word stems, which they complete with the first word coming to mind [written response]	Finite number of valid responses makes it simpler to classify answers as threatening or neutral based on pre-determined lists	Variations in frequency and word length influence stem-completion responses
Acoustical blend of word pairs	Acoustically blended emotional and neutral words are presented that differ by one phoneme. Participants select which word they heard from two displayed choices [keyboard or response box]	A greater number of novel stimuli previously unheard by participants	The selected response may not reflect the participant's initial resolution of ambiguity
**AMBIGUOUS IMAGES**			
Emotion recognition task with unaltered facial expressions	Participants classify emotional expressions presented [keyboard or mouse response]	Simple to design and administer even without the use of a computer	Prototypical expressions are easy to decode and may produce ceiling effects
Similarity rating task	Pairs of emotional stimuli of different intensities (e.g., moderately angry and very angry expressions) are presented, and participants rate the similarity of each pair on a numerical scale [keyboard or mouse response]	Measures relatively implicit processes and can be used with a variety of stimuli	It is difficult to ascertain precisely which features participants use to make their similarity comparisons
Emotion recognition task with morphed facial expressions	Participants classify emotional expressions presented. Response times may also be recorded [keyboard or mouse response]	A range of novel stimuli can be developed with different emotional intensities	At certain proportions and in certain combinations of emotions, morphed faces may appear unnatural and unlike those commonly viewed in everyday life
Incidental learning task	The learning phase presents facial expressions (e.g., positive, negative) in the center of the computer screen, which are predictive of the location of a subsequent target cue (i.e., negative faces predict upper targets, positive faces predict lower targets). The test phase presents neutral facial expressions followed by targets appearing with equal frequency at upper and lower locations. An interpretation bias is evident when, following neutral expressions, participants respond faster to target cues in the location predicted by specific faces. This task may also be used with morphed facial expressions during the test phase [keyboard or response box]	Measures relatively implicit processes possibly reducing demand characteristics and response biases. May also be used with other forms of ambiguous stimuli.	At certain proportions and in certain combinations of emotions, morphed faces may appear unnatural and unlike those commonly viewed in everyday life
Priming paradigm with morphed faces	Participants indicate the predominate emotion in morphed faces, which are presented following emotional or neutral primes (either words or images) [keyboard or response box]	Flexibility in exploring the impact of different types (e.g., linguistic or pictorial) and categories (e.g., pain-related, negative, positive) of primes and targets	At certain proportions and in certain combinations of emotions, morphed faces may appear unnatural and unlike those commonly viewed in everyday life. Prime words have different written and verbal frequencies, which should be considered
Priming paradigm with un-morphed faces	Cue-target pairs are presented, and participants indicate once they have determined which facial expression is presented. Speeded responses are expected when cue and target images are interpreted in the same manner [keyboard or response box]	Unlikely to be influenced by demand characteristics as participants do not explicitly state their interpretation of facial expressions.	It remains unknown how participants interpret both cue and target faces unless specifically asked
Rating tasks with blended faces	Different elements of the face are blended together to form ambiguous expressions (e.g., neutral eyes blended with a smiling mouth), which may be dynamically presented (i.e., an initial expression dynamically unfolds into a final expression). Participants rate each face on a specific dimension (e.g., trustworthiness) [keyboard or mouse response]	A range of static and dynamic novel stimuli can be developed from different prototypical emotional expressions	Depending on the combination of elements, blended faces may appear unnatural and unlike those commonly viewed in everyday life
Ambiguous visual scenes - Forced-choice paradigm	Forced paradigm task presents an ambiguous scene, and asks participants specific questions (e.g., for social anxiety research, “Does the scene show a popular or unpopular child?”) [keyboard or response box]	Complex scenes and situations can be depicted, including interactions between individuals. Possibly increased ecological validity compared to single faces and words	Static visual scenes are not wholly representative of dynamic interactions witnessed in everyday life. Responses may be subject to demand characteristics
Ambiguous visual scenes - Rating task	Following the presentation of an ambiguous visual scene, participants are asked to rate the image on a variety of dimensions, such as valence, arousal, judgment of danger, difficulty to tolerate, and how anxiety provoking the image was [keyboard or mouse response]	Complex scenes and situations can be depicted, including interactions between individuals. Possibly increased ecological validity compared to single faces and words	Static visual scenes are not wholly representative of dynamic interactions witnessed in everyday life. Responses may be subject to demand characteristics
Ambiguous visual scenes - Physiological arousal	During the presentation of an ambiguous image, data on physiological arousal (e.g., skin conductance response) is collected [recording of physiological arousal]	Complex scenes and situations can be depicted, including interactions between individuals. Possibly increased ecological validity compared to single faces and words. More objective response compared to subjective ratings	Static visual scenes are not wholly representative of dynamic interactions witnessed in everyday life. Verification needed as to whether physiological arousal truly does provide a valid and replicable measure of ambiguity resolution
**AMBIGUOUS SCENARIOS**			
Ambiguous scenarios test - Provide first explanation thought of	Following the presentation of an ambiguous scenario: - Participants write down the first explanation they think of [written response]	Open-ended responses do not constrain participants to a set of pre-determined interpretations	Responses may be subject to demand characteristics
- Imagine the outcome/state what is happening	- Participants write down the first explanation they think of [written response]	Open-ended responses do not constrain participants to a set of pre-determined interpretations	Responses may be subject to demand characteristics
- Rank order likelihood of possible interpretations	- Participants rank order a number of presented interpretations by their likelihood of coming to mind [written response]	Simple to administer without use of a computer	Closed response format limits participants to ranking pre-determined interpretations, none of which may reflect their own interpretation
- Rate level of agreement with possible interpretations	- Participants rate their level of agreement of number of presented interpretations [written response]	Simple to administer without use of a computer	Closed response format limits participants to rating pre-determined interpretations, none of which may reflect their own interpretation
- Rate likelihood of possible explanations	- Participants rate the likelihood of a number of presented interpretations [written response]	Simple to administer without use of a computer	Closed response format limits participants to rating pre-determined interpretations, none of which may reflect their own interpretation
- Imagine the scenario and rate its pleasantness	- Participants imagine the resolution of ambiguity and rate its level of pleasantness [written response]	Less influenced by demand characteristics as participants do not explicitly state their interpretation	Remains unknown exactly how participants interpreted the ambiguous scenario
- Imagine sentence and rate level of concern	- Participants indicate whether the event would cause them concern or not [written response]	Less influenced by demand characteristics as participants do not explicitly state their interpretation	Remains unknown exactly how participants interpreted the ambiguous scenario
- Alternative attributions measure	- Participants are encouraged to think of as many alternative interpretations to their initial interpretation, which are rated as positive or negative [written response]	Simple to administer without use of a computer	Although alternative interpretations are provided, responses may still be subject to demand characteristics
Ambiguous situations task	Following the presentation of an ambiguous scenario, participants answer a series of open-ended (e.g., What would you think in the situation?) and closed questions (e.g., Likert scale to rate the valence of the situation). This paradigm is similar to the ambiguous scenarios test but has been classified separately in this review for clarity [verbal and written response]	Simple to administer without use of a computer. Scenarios may be presented aurally so comprehension is not likely to be influenced by reading ability	The use of multiple response formats may be tiring and/or confusing for participants, especially children. Responses to open-ended questions may be subject to demand characteristics and influence responses to subsequent closed questions
Scrambled sentence task	Participants are presented with a series of words in random order that can be unscrambled and combined to form a negative or positive sentence [keyboard response]	Relatively simple to design and administer when comparing positive/negative interpretations. Flexibility for developing a large number of sentences. Demand characteristics can be reduced via used of cognitive load requirement	Potentially more difficult to develop appropriate stimuli for exploring anything other than positive/negative sentences
Word sentence association paradigm—traditional version	Participants are presented with a single word (benign or threatening) followed by an ambiguous sentence, and indicate whether the two are related or not. Response time may also be collected [keyboard or mouse response]	Flexibility in exploring the impact of different categories (e.g., negative, positive, disorder-specific) of primes and target sentences. Both self-report and response time data can be collected providing direct and indirect measures of bias	Unless comprehension questions included, cannot be certain participants read both sentence and words; they may respond to one or the other with no comparison made between the two
Word sentence association paradigm—modified version	Participants are presented with an ambiguous sentence followed by a single word (benign or threatening), and indicate whether the two are related or not. Response time may also be collected [keyboard or mouse response]	Flexibility in exploring the impact of different categories (e.g., negative, positive, disorder-specific) of primes and target sentences. Both self-report and response time data can be collected providing direct and indirect measures of bias	Unless comprehension questions included, cannot be certain participants read both sentence and words; they may respond to one or the other with no comparison made between the two
Lexical decision task - Grammatically correct	Participants read narrative texts with ambiguous sentences in which certain target words could be either threatening or neutral, and must indicate whether the target word is grammatically correct. Both accuracy and response times are recorded [keyboard or response box]	May measure relatively spontaneous and automatized interpretations (i.e., on-line interpretations at the time of reading)	Text readability may need consideration. Reading ability of participants may influence results, especially for more complex scenarios
- A real English word	Participants read narrative texts with ambiguous sentences in which certain target words could be either threatening or neutral, and must indicate whether the target word is a real English word. Both accuracy and response times are recorded [keyboard or response box]	May measure relatively spontaneous and automatized interpretations (i.e., on-line interpretations at the time of reading). Does not necessitate backwards checking of preceding text to provide answers	Text readability may need consideration. Reading ability of participants may influence results, especially for more complex scenarios
Reduced evidence of danger task	Children are presented with ambiguous and non-ambiguous spoken stories, and are instructed to determine as fast as possible whether the story was scary or not (i.e., have a good or bad ending). Specifically, children are asked after each sentence whether the story is scary. Children also indicate how they think each story will end, and how they would feel and think if in the situation [verbal response]	Individual differences in reading and writing ability do not affect participant responses; appropriate for children	May be subject to demand characteristics if children are informed that some of the presented stories are threatening
Sentence completion task - Complete with single word	Ambiguous sentences are presented with the final word missing. Participants complete the sentence with as many single-word responses that they can think of, and endorse the one response they believe best completes the sentence [written response]	Allows for testing of whether first interpretation provided is the one most endorsed	Unknown whether multiple responses occur prior to resolution of ambiguity or afterwards, the latter of which may reflect elaboration of responses based on demand characteristics of the task
- Complete with as many words as necessary	Incomplete ambiguous scenarios are presented, with participants providing a suitable ending in as many words as they like [written response]	Simple to administer without use of a computer	Responses may be subject to demand characteristics
Text comprehension task	Sentence pairs are presented, the first of which is ambiguous with both threatening and non-threatening interpretations. A continuation sentence is then presented corresponding to one of the two possible interpretations. Participants indicate whether the two sentences are related, with response times providing an indication of resolution of ambiguity [keyboard or response box]	Minimisation of demand effects as the critical measure is assessed covertly	Cannot be certain participants read each and every sentence, unless comprehension questions are added
Recognition task - Indicate whether recognize	Participants are presented with ambiguous sentences. In a subsequent recognition task, participants indicate whether or not they recognize disambiguated sentences [keyboard response]	Flexibility in exploring the impact of different scenario categories (e.g., pain-related, social threat)	Interpretation bias assessed retrospectively, so results may reflect negative recall bias
- Indicate similarity	Participants are presented with ambiguous sentences. In a subsequent recognition task, rate disambiguated sentences in terms of how similar they are to the original sentence in their meaning [written response]	Flexibility in exploring the impact of different scenario categories (e.g., pain-related, social threat)	Interpretation bias assessed retrospectively, so results may reflect negative recall bias
Reading time paradigm	An incomplete ambiguous sentence is presented with either threatening or non-threatening consequences. A disambiguating sentence is then shown which provides either a threatening or non-threatening conclusion to the scenario. Words are presented using a moving window technique, which are displayed when the participant presses a key. Reading time for target words are indicative of interpretation (faster for words congruent with interpretation of the ambiguous sentence) [keyboard reponse]	Allows for exploration of time-course of interpretation bias. Indirect measure of bias less influenced by demand characteristics	No guarantee participants fully read or engage with each sentence, unless comprehension questions are asked
Naming task	An incomplete ambiguous sentence is presented with either threatening or non-threatening consequences. A disambiguating sentence is then shown which provides either a threatening or non-threatening conclusion to the scenario. Words are presented one at a time. Naming time (i.e., verbal pronunciation) for target words are indicative of interpretation (faster for words congruent with interpretation of the ambiguous sentence) [verbal response]	Strategic vs. automatic biases may be explored by varying the interval between pre-target and target word. Indirect measure of bias less influenced by demand characteristics	No guarantee participants fully read or engage with each sentence, unless comprehension questions are asked
Ambiguous video clips - Participants rate how they would feel	Participants are shown video clips depicting a stranger approaching the camera and delivering ambiguous lines. Participants rate how they would feel in that situation [written response]	Increased ecological validity	Difficult to explore self-referent biases. Physical characteristics of actors may need to be controlled. Acting quality may influence believability of videos
- Participants rate clips	Participants are shown video clips of social interactions with ambiguous endings. Clips are rated in terms of valence, confidence in outcome, perceived escalation of threat, predictability and controllability [written response]	Increased ecological validity	Difficult to explore self-referent biases. Physical characteristics of actors may need to be controlled. Acting quality may influence believability of videos
- Participants rate possible explanations on their likelihood	Participants are shown video clips of social interactions with ambiguous endings, and subsequently rate the likelihood of positive, negative, and neutral explanations [written response]	Increased ecological validity	Difficult to explore self-referent biases. Physical characteristics of actors may need to be controlled. Acting quality may influence believability of videos. Closed response format limits participants to rating pre-determined interpretations, none of which may reflect their own interpretation
Confederate performing ambiguous behaviors	Participants perform a short speech, during which a confederate performs ambiguous behaviors (e.g., scratching head). Participants subsequently rate confederate behaviors using closed—and open-ended questions [written response]	Increased ecological validity, with participants directly exposed to ambiguous behaviors	Despite attempts at standardization, confederate behaviors may vary between participants. Confederate characteristics may also determine how noticeable ambiguous behaviors are
Story-stem paradigm	Children are presented with the beginning of an ambiguous story, and are then asked to complete the story using dolls and other props [behaviors are videoed and later systematically coded]	Suitable for children. Responses do not depend on reading ability	Characteristics of the experimenter and testing environment may influence child behaviors

## Review of available paradigms

For purposes of clarity, paradigms using different stimuli types are presented separately in this section, beginning with those using ambiguous words, followed by ambiguous images, and then ambiguous scenarios.

### Ambiguous words

#### Background

Numerous investigations exploring biased interpretation have made use of single ambiguous words presented individually to the participant, which are typically either homophones or homographs. Homophones are words which sound the same yet have different spellings and meanings (e.g., meat/meet, sea/see), while homographs are words which have identical spelling yet distinct meanings (e.g., bat—animal, wooden club; change—to alter, money; Drury, 1969; Gorfein and Weingartner, 2008). While some homographs are also homophones, this is not always the case (e.g., wound—injury, past tense of “to wind”). Homophones and homographs used in research studies have both threatening/emotional and neutral interpretations, and an interpretation bias is established as the number or proportion of each type of interpretation made by participants from a list of such words.

General advantages of using single words include their ease of administration, which may be either spoken or visual depending on whether homophones or homographs are used, and which may be presented without the use of a computer. A general limitation is that only a small number of appropriate words may be available depending on the population under study and the category of words required. For example, there are relatively few homographs that have both pain-related and neutral meanings (pain/pane being the most obvious homophone), therefore limiting their use in chronic pain research (Schoth and Liossi, 2016). When exploring biases in clinical populations, a further limitation is that disorder-relevant homophones are likely to have been heard and read more frequently by patients than healthy controls. To assess the impact of familiarity effects on participant responses, researchers can recruit health-care professionals along with healthy controls, exploring whether the former show increased biases relative to the latter. Due to their medical training health-care professionals may have a tendency to err on the side of caution however, and as such could demonstrate biased interpretations favoring clinical meanings for this reason also.

Normative data on the dominance of alternative word meanings are available for homographs (e.g., Nelson et al., 2004) and homophones (e.g., Gorfein and Weingartner, 2008), which are important to consider as certain interpretations are more likely than others in the absence of context. These have not always been consulted by researchers when selecting their stimuli however. It should also be noted that the relative dominance of alternative word meanings is likely to vary across time, culture, and geographic region, and therefore normative data may be more applicable to certain populations than others (i.e., populations similar to the samples from which normative values were obtained). Presentation time of ambiguous words is also an important consideration, particularly for visually presented homographs which in some studies are presented in list format.

#### Homophone tasks

The homophone task is a commonly used paradigm which presents participants with spoken homophones that have both threatening and neutral associations (e.g., die/dye, pain/pane, slay/sleigh) which they must write down. Studies using this paradigm have explored interpretation biases in a range of populations, including individuals with high anxiety (Mathews et al., 1989b; Mogg et al., 1994), depression (Mogg et al., 2006), and chronic pain (Pincus et al., 1996). The homophone task may be modified for children, presenting two pictures after the spoken homophone corresponding to their two possible interpretations (e.g., Bury/Berry—coffin picture/fruit picture), with the participant selecting (i.e., verbally stating or pointing to) the picture best matching the word (e.g., Hadwin et al., 1997). This version has been infrequently used in the literature however, and further exploration in a range of populations is needed to ascertain its usefulness. A strength of the pictorial homophone task is that individual differences in reading and writing ability do not influence participant responses. An overall strength of both versions of the homophone task is that they are simple to administer, not requiring the use of a computer, and at a minimum necessitate only pen and paper. A general limitation is that threatening and neutral associations of the same homophone often have different written and verbal frequencies of use, which may therefore influence participant responses (Simpson and Krueger, 1991).

#### Lexical decision task

Homographs with threatening and neutral meanings (e.g., terminal, growth, beat) have frequently been used in differing variants of the homograph task, often as primes. Richards and French (Richards and French, 1992) used homographs as primes in a lexical decision task, which are presented individually on a computer screen and followed by a target letter string (either a real word or a non-word). Real-words are either related to the threatening (e.g., arms-weapon) or non-threatening (e.g., arms-legs) meaning of the prime. Participants indicate via keyboard or response box whether the target is a real-word or not, with the notion that faster response times to negative compared to neutral words are indicative of a priming effect for that meaning. Numerous other studies have explored biased interpretations using the lexical decision task, including those exploring biases in trait rumination (Mor et al., 2014), depression (Bradley et al., 1995), and pain anxiety (Vancleef et al., 2016). The paradigm may also be adapted to present homographs aurally as opposed to homographs visually (Chapman and Martin, 2011).

Different stimulus onset asynchronies SOAs (i.e., the time between the presentation of the homographic prime and the target) may influence the pattern of results found (Richards and French, 1992; Chapman and Martin, 2011; Jalal and Amir, 2014), suggesting attentional factors to also influence patterns of bias in the lexical decision task. Overall, as the lexical decision task provides an indirect measure of interpretation biases based on response times, an advantage of this paradigm is that responses are less likely to be influenced by demand characteristics compared to paradigms where participants must state their interpretations verbally or in writing. While is it sometimes assumed participant decisions are without internal noise however, research shows this to be untrue; response times for low-frequency target words in particular have been shown to be influenced by participants guessing responses (Diependaele et al., 2012).

#### Homographic response task and sentence generation task

In the homographic response task (this paradigm is referred to by numerous names, including the “ambiguous cues task” and “single-word associate homographic response task”) participants are presented with homographs, and asked to write the first word they think of related to it. Independent raters subsequently classify the response words as threat-related or benign/neutral. This paradigm has been used to explore biases in many populations, including patients with chronic pain (Pincus et al., 1994; McKellar et al., 2003), and chronic fatigue syndrome (Moss-Morris and Petrie, 2003) [it should be noted in this instance words were presented verbally, two of the 15 being homophones–vein/vain, weak/week]. A variant of this task requires participants to provide multiple associated words, which are again rated for their content (Jelinek et al., 2009). Similar to the homographic response task, the sentence generation test presents participants with a homograph, and asks them to form a short sentence including that homograph. These response sentences are subsequently rated as either threat-related or neutral (Taghavi et al., 2000). Overall, homographic response and sentence generation tasks are both relatively simple to administer, often without the use of a computer, and have frequently been adopted in the literature. Responses may be subject to demand characteristics however, and a participant's provided response(s) may not necessarily represent the first that came to mind; this may be especially true if participants are provided unlimited time to make their response. Researchers should also be aware that, even though homographic words have multiple associations, certain associations may be more dominant than others (i.e., more frequently reported, as index by normative data), and some homographs have more associations than other homographs (Nelson et al., 2004). These factors are essential to consider when designing lists of appropriate stimuli, especially when attempting to compare between different categories of words. Furthermore, as with all paradigms which require classification of participant responses, a degree of subjectively may exist as responses themselves can be ambiguous and therefore multiple, blind raters are required.

#### Word stem completion task

The word stem completion task presents participants with three-letter word stems, and requires them to complete the stem with the first word coming to mind which may be either threatening or neutral (e.g., ache, achieve; Edwards and Pearce, 1994; Griffith et al., 1996) [It should be noted that this task has also been used as part of research exploring implicit memory biases in patients with anxiety and post-traumatic stress disorder disorders (Mathews et al., 1989a; Zeitlin and McNally, 1991), although patterns of interpretation have not always been assessed]. Like the homographic response and sentence generation test, the word stem completion task requires independent judgment as to whether participant responses fall into emotional/threatening or neutral categories. A degree of subjectivity may exist in this procedure. For example, considering the chronic pain literature, certain words participants respond with are homographs with both positive and pain-related connotations (i.e., tender-pain, tender-gentleness; sharp-pain, sharp-clever). Characteristics of the raters may potentially influence the results, and therefore multiple, blind, independent raters should be used to minimalize such issues. Compared to the other two paradigms, an advantage of the word stem completion task is that there are a finite number of possible completions per stem, which may be determined in advance along with their classification into emotional or neutral categories. Participant responses to the word stem completion task have been shown to be influenced by word frequency and length however (Mueller and Thanasuan, 2014).

#### Acoustical blend of words

Ambiguous auditory stimuli may also be used to explore interpretation biases. In this paradigm, participants are presented with acoustically blended emotional and neutral words that differ by one phoneme (e.g., joy-boy; sad-sand; hated-heated), and must then select using keyboard or response box which word they heard from two displayed choices. A number of studies using this paradigm have explored interpretation of depressotypic–neutral word blends (Dearing and Gotlib, 2009; Masland et al., 2015), although overall this novel task has been used much less frequently than the others involving single ambiguous words, possibly due in part to the additional time and skills required to create the stimulus set. An advantage of this paradigm is that a greater range of novel stimuli may be developed when compared to using homophones and homographs. A limitation is that responses may be influenced by differences in auditory processing skills, along with individual differences in hearing ability necessitating the use of a sound attenuated room. It should also be borne in mind that the blended words are of course unnatural. Furthermore, in this paradigm the selection of one word from two possible choices does not necessarily mean the selected word matches the participants own resolution of ambiguity, as they may have interpreted the word differently. Factors such as word frequency may also influence participant responses, and should be carefully controlled for when developing such stimuli.

### Ambiguous images

#### Background

A number of studies have explored interpretation biases using ambiguous images, often with emotional and neutral faces. General advantages of using images include a much greater availability of disorder-relevant stimuli when compared to either homophones or homographs (e.g., angry, sad, and pain faces may be used for exploring biases in anxious, depressed, and pain populations respectively). Related to this, researchers are able to develop and use novel images which have not been seen before by participants, thus helping to overcome familiarity effects which may be problematic when using ambiguous words. Furthermore, unlike a number of ambiguous word paradigms, independent raters are not required to judge participant responses as falling into either threatening/emotional or neutral categories, as the participant indicates this themselves. While this reduces potential biases attributed to characteristics of the raters from influencing the results, independent raters are often still used in the development and classification of the stimuli. Multiple independent raters should therefore be used in the classification of pictorial stimuli into their emotional categories. Another general limitation is that facial expressions in real life are dynamic, and therefore interpreting an ambiguous static expression is likely to be different to interpreting an ambiguous dynamic expression. Furthermore, interpretation of facial expressions in real-life is influenced by contextual information stemming from the environment and individual's body language (Wieser and Brosch, 2012; de Gelder et al., 2014; Schoth et al., 2015; Kim and Lee, 2016), which is not captured in a single isolated expression.

#### Emotion recognition task and similarity rating task

The emotion recognition task presents (typically via computer) unaltered facial expressions including neutral expressions to participants, asking them to simply identify the emotion displayed (e.g., Winton et al., 1995; Maniglio et al., 2014). Although relatively easy to implement, use of prototypical expressions which are easy to decode may result in ceiling effects (Gebhardt and Mitte, 2014). An alternative paradigm is the similarity rating task, which presents pairs of emotional expressions of different intensities (e.g., moderately angry to very angry), and asks participants to rate the similarity of each pair on a numerical scale. If, for example, anxious individuals misinterpret ambiguous emotional information, moderate and very angry images should be interpreted as more similar than by non-anxious individuals [moderate images may also be interpreted in a non-threatening or neutral manner]. This paradigm has been commonly used across a range of research, with the advantage that it does not directly instruct participants to interpret the presented information, and therefore may measure relatively implicit processes (Gebhardt and Mitte, 2014). A variety of stimuli may also be used, including facial expressions only, but also images of the entire body which therefore provide information on body language. Gebhardt and Mitte (2014) used whole body photographs of male actors displaying angry, happy, and neutral expressions with a naturalistic variability of low to high intensity. Paired images of different models are presented, followed by a Likert scale requiring participants to indicate how similar the two models are. While whole body images are more naturalistic than isolated facial expressions, a general limitation with complex images is a difficulty ascertaining precisely which features participants are using to make their similarity comparisons.

#### Emotion recognition task with morphed facial expressions

As opposed to using unaltered facial expressions, in recent years an increasing number of studies with a variety of populations have interpolated, or morphed, facial expressions with one another (e.g., angry and neutral expressions) to various proportions (e.g., 90% angry/10% neutral; 50% angry/50% neutral), thereby increasing the ambiguity of such images to varying degrees (e.g., Jhung et al., 2010; Jusyte and Schönenberg, 2014). Such expressions are presented to participants via computer in a modified version of the emotion recognition task, who judge the predominate expression present, thereby indicating their resolution of ambiguity. In addition to these classifications, participant response times may also be recorded with the assumption that should negative interpretations be more accessible to the individual's mind, these will be faster than positive or neutral interpretations (Maoz et al., 2016). To provide an example, Liossi et al. (2012) used ambiguous facial expressions to explore biases in mothers of children with chronic abdominal pain and mothers of healthy, pain-free children. Facial expressions of pain were interpolated with sad, angry, fearful, happy, and neutral faces in various different proportions. Overall, an advantage of the emotion recognition task with morphed facial expressions is that range of novel stimuli can be developed with different emotional intensities.

A limitation of this paradigm is that morphed images may at certain proportions and in certain combinations of emotions appear unnatural and unlike those commonly viewed in everyday life however. Mechanisms underlying the integration of facial features into a holistic perceptual unit are relatively unknown (Curby et al., 2013). Another limitation of this paradigm is that, unless specifically investigated, it also remains unknown whether for different combinations of facial expressions and different proportions of morphing, participants use different sources of information when resolving the ambiguity. Cultural factors (Blais et al., 2008; Engelmann and Pogosyan, 2013) and sex (Sawada et al., 2014) have also been shown to shape emotional perception and responses to emotional faces.

#### Incidental learning task

The incidental learning task is a computerized paradigm which requires participants to detect the location of a target following a cue (e.g., a facial expression). Participants are unaware that the location of the target is contingent on the valence or category of the cue. Should learning occur, participants will be faster to detect targets in the location predicted by the cue. The inclusion of an ambiguous cue can therefore be used to explore interpretation biases via target response times. An example will serve to clarify the experimental procedure. Yoon and Zinbarg (2008) presented negative and positive faces during the learning phase in the center of the computer screen, which were predictive of the location of a subsequent target cue (i.e., upper or lower). The test phase included neutral faces followed by targets appearing in upper and lower locations with equal frequency. A negative interpretation bias is evident when, following neutral faces, participants respond faster to targets in the location predicted by negative faces. In contrast, a positive interpretation bias is evident when, following neutral faces, participants respond faster to targets in the location predicted by positive faces. An advantage of the incidental learning task is the measurement of behavioral response patterns, which may therefore reduce the effects of demand characteristics or response biases (Yoon and Zinbarg, 2008; Khatibi et al., 2014). This paradigm may also be used with morphed facial expressions; for example recent studies have implemented morphed painful and happy expressions at a 50%:50% proportion (Khatibi et al., 2014, 2015). A limitation of using morphed faces however is that these may appear unnatural and unlike those viewed in everyday life.

#### Priming paradigm with morphed and un-morphed faces

Ambiguous facial expressions have been used in priming paradigms, investigating whether interpretation of a stimulus may be primed by negative or positive information. When featuring morphed facial expressions, participants are instructed to indicate the predominate emotion in a target. For example, Grynberg and Maurage (2014) asked healthy participants to determine the predominate emotion in a series of 50%:50% morphed faces (i.e., pain/neutral, pain/happy, pain/fearful, fearful/neutral, fearful/happy, happy/neutral). During each trial a single prime word, either negative (e.g., discouragement), distress (e.g., worried), empathic concern (e.g., tender), or neutral (e.g., salute), was presented subliminally for 25 ms, followed by the ambiguous facial expression for 750 ms. As with other paradigms using morphed faces, a limitation is that these may appear unnatural.

An alternative version of the priming paradigm involves the use of un-morphed faces, wherein participants must simply indicate once they have determined the facial expression. Yoon and Zinbarg (2007) used angry, disgust, happy, and neutral faces in cue-target pairs. For each pair, participants were required to indicate via manual response once they had determined what facial expression each image was depicting. The underlying premise is that response times for the target image will be influenced by the participant's interpretation of the cue image; speeded responses are expected when cue and target images are interpreted in the same manner due to priming effects of the first image. An advantage of this variant is the elimination of potential effects of response selection bias, as participants are not required to indicate what emotion they interpret the images as projecting, but rather that they have simply made their interpretation. Of course, the counter-argument is that researchers are therefore unaware how participants interpret both cue and target faces. On certain trials, however, participants were required to provide a short story linking both images and indicating how the people were feeling, thus also providing more direct information on stimuli interpretation. Overall, an advantage of both versions of the priming paradigm is the flexibility offered in exploring the impact of different types (e.g., linguistic or pictorial) and categories (e.g., pain-related, negative, positive, neutral) of primes and targets. The paradigm may therefore be modified for use with a range of clinical and non-clinical populations, and the specificity of bias explored in depth.

#### Rating tasks with blended faces

An alternative method to morphing different emotional expressions is to combine different elements of the face to create ambiguous blended expressions, for example a smiling face with happy or non-happy eyes (e.g., happy eyes and neutral mouth, neutral eyes and smiling mouth). Using this blending approach to explore biases in social anxiety, Gutiérrez-García and Calvo (2016) created blended faces in motion (i.e., 2000 ms video clips). An initial expression shown for 500 ms (e.g., neutral face) dynamically unfolded over 1000 ms into a final expression shown for 500 ms (e.g., happy face). The task of the participant was to rate the trustworthiness of each face. Many different combinations of blended faces may be created using a variety of expressions, with the authors in a second experiment blending smiling lower faces with neutral, surprised, fearful, sad, disgusted, and angry upper faces. Many possibilities exist for future research adapting this paradigm, as a range of ambiguous stimuli can be developed from prototypical emotional expressions which would enable the exploration of interpretation bias specificity. Depending on the combination of elements blended images may appear unnatural however, and may not reflect those seen in everyday life.

#### Ambiguous visual scenes–forced-choice paradigm, rating tasks and physiological arousal

Ambiguous visual scenes contain contradictory or ambivalent information pertaining to the situation (Kirschner et al., 2016), which have been used in a limited number of interpretation bias paradigms. Forced-choice paradigms require participants to choose between two or more possible interpretations of an ambiguous scene. For example, In-Albon et al. (2009) explored disorder-specific biases in children with separation anxiety disorder (SAD), social phobia, and healthy controls. Participants were required to specify via button press whether the visual scene depicted an arrival or departure situation, or a popular or unpopular child. Participant responses to ambiguous scenes provides an indication of interpretation bias.

Further to participants making forced choices, interpretation biases can also be explored via rating tasks and measures of physiological arousal. As part of an experimental design exploring intolerance of uncertainty and ambiguity, Kirschner et al. (2016) presented ambiguous visual scenes which participants subsequently rated on several dimensions, including valence, arousal, judgment of dangerosity, difficulty to tolerate, and how anxiety provoking the image was. This study also collected skin conductance data. Measures of physiological arousal may provide a more objective indication of ambiguity resolution (i.e., arousal will be higher for ambiguous images interpreted as threatening compared to those interpreted as non-threatening). However, further research is clearly needed to help fully establish the advantages of using such measures relative to collecting subjective ratings.

Overall, few studies have used ambiguous scenes relative to facial expressions alone, and many possibilities for the development of novel paradigms exist. An advantage of visual scenes is that more complex situations and behaviors can be depicted than is possible using facial expressions alone, including social interactions between individuals. A limitation is that visual scenes may require more time to develop and match. Visual gaze and attention are influenced by the low-level features of the visual scene (Henderson, 2003; Torralba et al., 2006), along with properties of valence and arousal (Fernandes et al., 2011; Ni et al., 2011). Attention, in turn, influences processes of interpretation (Neisser, 1967; Pomplun et al., 1996; Fu et al., 2006). It is therefore critical that research using visual scenes assess, control, and provide details on low-level features, valence and arousal (Nummenmaa et al., 2006; Schoth et al., 2015), especially when comparing participant responses across multiple stimuli conditions (e.g., ambiguous vs. non-ambiguous conditions). It should also be noted that visual scenes are static representations of dynamic interactions, and in real-life human behaviors may serve to either increase or decrease ambiguity.

### Ambiguous scenarios

#### Background

Events in everyday life are complex, and therefore interpretation of single ambiguous words or images may to a certain extent lack ecological validity (Hirsch and Mathews, 1997). Further to ambiguous visual scenes discussed above, researchers have also used ambiguous scenarios and vignettes (including ambiguous SMS text messages, Kingsbury and Coplan, 2016) to explore interpretation biases in a variety of tasks. Examples of scenarios used in former research include “*You have visitors round for a meal and they leave sooner than expected*” and “*You are talking to an acquaintance who briefly looks out of the window*” (Stopa and Clark, 2000), although scenarios may be longer than single sentences. An advantage of using scenarios, compared to single words or images, is that much more detailed stimuli can be created and tailored to the population under study. While the psychometric properties of scenarios have been explored (for example, the ambiguous scenarios test for depression developed by Berna and colleagues reported a Cronbach's alpha of 0.82 Berna et al., 2011), scenarios are infrequently matched in terms of readability, and it is possible that certain scenarios may be more easily understandable and/or more ambiguous than others.

#### Ambiguous scenarios test

Ambiguous scenarios are often used in variations of the ambiguous scenarios test, although researchers do not always refer to their paradigm by this name. These tests are often presented on paper, with participants providing their responses in writing. Task requirements vary, depending in part on the population under investigation, with some studies open-ended and requiring participants to write down the first explanation they think of for the scenario (e.g., Stopa and Clark, 2000), some requiring participants to write down the imagined outcome or what they think is happening (e.g., Waite et al., 2015; Orchard et al., 2016), some to rank the order of multiple interpretations in terms of the likelihood each would come to their mind (e.g., Amir et al., 1998), some to rate their level of agreement of possible interpretations (e.g., Constans et al., 1999) or the likelihood of possible interpretations (Wenzel and Lystad, 2005), and some to imagine the scenario outcome and rate its pleasantness (e.g., Berna et al., 2011) or the participant's own level of concern (e.g., Davey et al., 1992). Such studies have been conducted with a range of populations, including individuals with heightened anxiety, social phobia, and depression.

Some studies using ambiguous scenarios collect multiple measures. Heathcote et al. (2016) required participants to rate whether each interpretation was likely to pop into their head, select the interpretation that most easily popped into their head, and finally rate their belief that each interpretation would actually happen in reality. Ewing et al. (2016) asked children what they thought was happening in each ambiguous situation and then asked them to rate which one of two presented interpretations was most likely. An alternative paradigm, the alternative attributions measure, records the number of alternative interpretations participants are able to make, and whether these are positive or negative in nature (Berry and Cooper, 2012). Overall, a general advantage of the ambiguous scenarios test is that all versions may be presented without the use of a computer, using nothing more than pen and paper. Versions featuring open-ended responses do not constrain participants to a set of pre-set interpretations, although responses may be subject to demand characteristics. A limitation of rating pre-set interpretations is that none may necessarily reflect the participant's personal interpretation of the ambiguous scenario however. An advantage of asking participants to indicate levels of pleasantness or concern is that they do not have to explicitly state their interpretation of the event, and therefore may be less subject to demand characteristics. It will remain unknown exactly how participants interpreted the ambiguous scenario however. Considering this, it may be most advantageous for researchers using the ambiguous scenarios test to adopt open-ended responses followed by ranking/rating of specific interpretations (Stopa and Clark, 2000) or forced choice questions (Field and Field, 2013), thereby collecting multiple measures.

#### Ambiguous situations task

The ambiguous situations task is largely similar to the ambiguous scenarios test, although collects multiple open and closed responses differing in wording to those reported above for the ambiguous scenarios test. For purposes of clarify the ambiguous situations task is therefore presented separately here in this review. Using this paradigm, Farrell and colleagues presented ambiguous scenarios to children with obsessive-compulsive disorder and their mothers, followed by multiple open-ended questions (i.e., “What would you think in the situation?,” “What would you feel in the situation?,” and “What would you do in the situation?”) and closed questions (i.e., Likert scales are used to rate the valence of the situation, along with ratings for questions “How confident are you that you could cope with this situation?” and “If you were in this situation how difficult would you find it?” “What would you feel if you were in this situation?” is also asked, with participants indicating whether they endorse a series of possible feelings). Data from open-ended questions are subsequently coded by multiple coders (Farrell et al., 2015). An advantage of this paradigm is that it is relatively simple to administer, with scenarios provided aurally for children ensuring comprehension is not influence by reading ability. A potential limitation is that the use of so many responses per scenario may be tiring or confusing for children. Furthermore, open-ended questions were asked first which may be subject to demand characteristics and potentially influence responses to closed questions.

#### Scrambled sentence test

The scrambled sentence test (Wenzlaff and Bates, 1998) presents participants with a series of words (e.g., *looks the future bright very dismal*) that can be unscrambled and rearranged to form a coherent sentence with either a positive (i.e., *the future looks very bright*) or negative (i.e., *the future looks very dismal*) meaning. Specifically, during each trial the computerized version first presents the scrambled sentence which participants are instructed to mentally unscramble and form a grammatically correct and meaningful statement using five of the six words. Once they have indicated doing this, a second screen presents each word alongside a number; participants use the numbers to report their unscrambled sentence. Interpretation bias is inferred based on the number of negative and positive interpretations made.

This paradigm has most often been used to explore biases in depression when comparing positive/negative interpretations (e.g., Everaert et al., 2013, 2014, 2016). An advantage of this paradigm is that it is relatively simple to design and administer, and as neither homographs or homophones are required (of which there are a limited number for any participant population) a large number of sentences can be developed for testing purposes. A limitation is that responses may be subject to demand characteristics, as it may be relatively easy to guess the underlying study hypothesis. In order to overcome this, the paradigm may be described as a language experiment, and a cognitive load component is typically added which requires participant to memorize a six-digit number prior to the experimental block that is reported after the block. These strategies, as well the use of numbers to report the word order for each sentence, aim to reduce demand characteristics such as social desirability from influencing participant responses. A further limitation is that it may be difficult using this paradigm for exploring anything other than positive/negative interpretations while keeping the experimental aims hidden from participants yet designing plausible stimuli, although it is still possible to explore negative/neutral and positive/neutral interpretations.

#### Word sentence association paradigm–traditional version

The traditional version of the word sentence association paradigm presents during each trial a single prime word followed by an ambiguous sentence. Participants indicate via keyboard whether or not the word is related to the ambiguous sentence. For example the sentence “*People laugh after something you said*” follows either the threatening prime “*Embarrassed*” or the benign prime “*Funny*.” Similar to priming paradigms with single words and images, an advantage of the word sentence association paradigm is the flexibility offered in exploring different categories of primes and sentences, and the ability to collect both direct (participant responses) and indirect (response times) measures of bias. A potential limitation is that it is unknown if participants really do read each sentence presented and compare these to the single words, or whether they respond to the single words only (Beard and Amir, 2009). One way to overcome this is to include comprehension questions.

#### Word sentence association paradigm–modified version

The modified version of the word sentence association paradigm is similar to the traditional version, although each trial presents the ambiguous sentence before the word (e.g., Cowden Hindash and Amir, 2012; Cowden Hindash and Rottenberg, 2017). For example the sentence “*People always tell you to smile”* proceeds the benign word “*Loved”* or the negative word “*Defective.”* The task of the participant remains to judge as quickly as possible whether the word is related to the sentence. Unlike the traditional version, the modified version cannot be considered a priming task however (this is important to consider when comparing the results of these different versions). Rather, the participant's resolution of ambiguity influences their subsequent judgment of the word. As with the traditional version, strengths of the modified version include flexibility of stimuli categories and the collection of both direct and indirect measures (participant responses and response times respectively). A limitation is that, unless comprehension questions are included, it is not guaranteed participants actually read each sentence and engaged with the task as intended.

#### Lexical decision tasks

Reaction times are recorded in lexical decision tasks, which feature narrative texts with ambiguous sentences in which certain target words could be either threatening or neutral. Depending on the task instructions, participants must indicate whether the target word is (i) grammatically correct or (ii) a real word, with the assumption that participants respond faster to words that are congruent with their concerns or fears. Research using this paradigm has explored biases in a range of populations, including individuals anxious about interviews (Hirsch and Mathews, 1997), and individuals with social phobia (Hirsch and Mathews, 2000) and pain-related anxiety (Vancleef et al., 2009). An advantage of such paradigms is the flexibility available for stimuli development, and also the argument that such tasks may measure more automatic and spontaneous interpretations (i.e., on-line interpretations made while reading). That is, if participants make inferences while reading incomplete sentences, responses should be faster to target words matching this inference. However, it has been noted that participants are less likely to make backward checks to the preceding text when instructed to indicate whether the probe word is a real word or not, compared to whether the word is grammatically correct (Hirsch and Mathews, 1997). As such, the latter may be more reflective of on-line interpretations than the former.

### Reduced evidence for danger task

The reduced evidence for danger (RED) bias task is an infrequently used paradigm which presents spoken ambiguous scenarios, with children instructed to indicate as soon as possible if the story will have a bad or happy ending. That is, after each sentence children indicate whether the story is scary (bad ending) or non-scary (good ending; e.g., Muris et al., 2000). An advantage of this paradigm is that it may be used not only with children, but all age groups. As scenarios are spoken (and typically presented via audiotape to eliminate variation in tone and inflection), results will not be affected by variations in reading ability. Responses may be subject to demand characteristics if participants are informed that some of the presented stories are threatening however.

### Sentence completion tasks

In the sentence completion task, participants must resolve ambiguous sentences (e.g., “*As you give a speech, you see a person in the crowd smiling, which means your speech is___*”) with as many single-word responses that they can think of, and endorsing the one response they believe best completes the sentence (e.g., Huppert et al., 2007). Similar to this, another method is to present incomplete ambiguous scenarios, asking participants to provide a suitable ending in as many words as they like (e.g., Woud et al., 2014). An advantage of both versions of the sentence completion task is that they are simple to administer, requiring only pen and paper. Responses may be susceptible to demand characteristics however, and also require multiple, blind and independent raters to classify participant responses. Should raters not be blind to participant groupings, and should responses themselves be ambiguous, classification of responses may be biased.

### Text comprehension task

The text comprehension task (MacLeod and Cohen, 1993) presents participants with sentence pairs, the first of which is ambiguous with both threatening and non-threatening interpretations (a cue word may be initially presented to disambiguate this sentence, which serves as a comparison condition). A continuation sentence is then presented corresponding to one of the two possible interpretations. The underlying principle is that continuation sentences which correspond to the participant's interpretation of the initial sentence will be read faster, and therefore reading speed (or comprehension latency) of such sentences provides an index of resolution of ambiguity. Participants press a button after reading each sentence, which therefore provides an measure of reading time. Advantages of this paradigm include minimization of demand effects as the critical measure is assessed covertly, and also the fact that reading times of sentences are considered more naturalistic compared to paradigms involving response times to single ambiguous words (Mogg et al., 2006). A limitation is that it cannot be certain participants read each and every sentence unless comprehension questions are added.

### Recognition tasks

An increasing number of studies are exploring biased interpretations via memory recognition tasks. Participants are presented with ambiguous scenarios with both threatening and non-threatening interpretations. During a later recognition test, they must determine via keyboard response whether derivatives of the scenarios correspond to the same meaning as that scenario formally presented (e.g., ambiguous scenario “*All the guests at the wedding giggled at Mark's speech*”; threatening derivative “*All the guests at the wedding ridiculed Mark's speech*”; non-threatening derivative “*All the guests at the wedding enjoyed Mark's speech*”; from Gannon and Rose, 2009). The underlying notion is that ambiguous stimuli are implicitly interpreted in a manner consistent with one's cognitive schemas, which influences subsequent recognition. Using a slightly different method, Williamson et al. (2000) presented ambiguous body-related and ambiguous health-related scenarios auditorily to participants with eating disorders, body dysmorphic disorder, and nonsymptomatic controls. Participants were required to listen to each scenario, and imagine themselves in the situation. Disambiguated sentences were then presented in random order, including thinness and fatness-related interpretations for ambiguous body-related scenarios, and healthy and unhealthy interpretations for health-related scenarios. In this task, participants had to rate the similarity of each disambiguated sentence to the former scenarios heard.

Exploring biases in individuals with high and low cancer fear, Miles and colleagues presented participants with an ambiguous scenario in which the final word was fragmented (e.g., “*Your surgeon says he can tell instantly from the position of your tumor whether surgery is p_ssible*”). Participants completed the word fragment, and then answered a comprehension question, both of which were implemented to ensure they had read and understood the scenario. Following all scenarios, participants were then presented with test sentences, including positive and negative interpretations of the former scenario, and asked to rate the similarity of each sentence to the scenario (Miles et al., 2009). Overall, an advantage of using recognition paradigms is that interpretation biases may be assessed without directly asking participants, a factor of importance when assessing topics wherein responses are likely to be influenced by social desirability concerns (Blake and Gannon, 2014). A limitation, however, is that results may reflect a negative recall bias as opposed to an interpretation bias.

### Reading time paradigm and naming task

The reading time paradigm has been used as a measure of inference processing. Specifically, Calvo et al. (1997) presented incomplete ambiguous sentences with either threatening or non-threatening consequences (e.g., “*At night the old woman was crossing the motorway when a lorry approached her at high speed*”). A disambiguating sentence is then shown which provides a threatening (e.g., “*The lorry knocked down…*”) or non-threatening (“*The lorry braked…*”) conclusion to the scenario. Participants read the sentences, with words presented in a moving window of 1 to 4 words, with participants progressing though the sentence via button press. Reading time for specific target words, which either confirm or disconfirm the consequence previously implied in the ambiguous sentence (i.e., “*knocked down*” and “*braked*” in this example), are taken as an index of interpretative bias. Similar in design to the reading time paradigm is the naming task (Calvo and Castillo, 1997), which presents each word individually at a pre-determined pace. The target word is embedded within the disambiguating sentence (flanked by asterisks to identify it), which resolves the ambiguity in either a threatening or non-threatening manner. Participants must pronounce the target word as quickly as possible, which is recorded by microphone. Naming time of target words provides an index of resolution of ambiguity. Exploration of strategic vs. automatic biases may achieved by varying the interval between pre-target and target word (i.e., 500 ms and 1250 ms respectively. Overall, an advantage of both reading time paradigm and naming task is that they do not directly ask the participant for their resolution of ambiguity, and therefore are less susceptible to demand characteristics. As with other response time tasks however, there is no guarantee participants have fully read each sentence unless comprehension questions are asked, and participant typical reading speeds may also need to be considered.

### Ambiguous video clips

Video clips and vignettes have also been used to explore interpretation biases, although less frequently than written scenarios. Resolution of ambiguity may be determined by asking participants how they would feel in ambiguous situations. Amir et al. (2005) showed short video clips that featured a stranger approaching the camera and delivering positive (e.g. “*I really like your shoes*”), negative, (e.g., “*That is a horrible hair cut*”) and ambiguous lines (e.g., “*That is an interesting shirt you have on*”), with the task of the participant to rate how they would feel in that situation. An alternative method to collecting ratings on participants' own feelings is to collect ratings on the ambiguous video clips themselves. Elwood et al. (2007) showed participants short threatening, positive, and neutral video clips of social situations with ambiguous endings, who were then required to rate each clip in terms of valence, confidence in outcome, perceived escalation of threat, predictability and controllability. Victims of interpersonal trauma, relative to non-victims, rated threatening clips as more predictable and as increasing more in risk. A further possibility is to ask participants to rate different explanations for ambiguous video clips. Wenzel et al. (2005) showed participants videos of positive, negative, and neutral situations. Participants were subsequently questioned on certain factual details of the videos (e.g., “*At 9:00 p.m., the woman looked at her watch and told the man she needed to go because…”*), and asked to rate the likelihood of possible positive, negative, and neutral explanations. Participants were also asked “What will happen next?” (although responses from this question were not actually used in the analysis, yet represent a possibility for future studies). Overall, an advantage of using video clips is that they may possess higher ecological validity than written text or visual scenes, especially when depicting social interactions. However, it is difficult to explore self-referent vs. other-referent biases using pre-recorded videos (unlike written text, which can written in the first person, e.g., “*You walk into a room filled with strangers…”*), and factors such the physical characteristics of the actors need to be controlled for, especially if exploring different categories of interactions. Acting quality is also an important consideration, and may influence believably of the scenarios.

### Confederates performing ambiguous behaviors

Although infrequently used, interpretation biases may be explored in relation to ambiguous social behaviors performed by confederates of the researcher. Kanai et al. (2010) asked participants to perform a short speech to a confederate, who performed ambiguous behaviors (e.g., clearing throat, running fingers through hair, scratching head). After the speech, participants answered closed- and open-ended questions regarding the confederate's behavior. An advantage of this paradigm using confederates of the researcher is high ecological validity, as participants are directly placed in a situation with ambiguous behaviors. A limitation is that despite attempts to standardize confederate behaviors, variation in performance may occur for different participants or across different conditions (e.g., ambiguous vs. non-ambiguous situations) thus affecting results.

### Story-stem paradigm

The story-stem paradigm is a novel approach for assessing interpretation biases in young children. During this task, children are presented with the beginning of an ambiguous story, and are then asked to complete the story using dolls and other props. Their behaviors are filmed and later systematically coded (e.g., Dodd et al., 2012). A strength of this paradigm is that results do not depend on individual reading ability. Characteristics of the experimenter and testing environment may influence child behaviors however, and so should be carefully considered and standardized across participants.

## Discussion

The aim of this article was to provide a review of experimental paradigms available for exploring biased interpretation of ambiguous information with both threatening/emotional and neutral interpretations. Forty-five paradigms were identified, including eight using single ambiguous words, 10 using ambiguous images, and 27 using ambiguous scenarios. Researchers wishing to explore biased interpretations are therefore faced with a large choice of paradigms, each with their associated strengths and limitations. Here we offer general recommendations for researchers which is hoped will be useful when designing future studies.

### Stimuli validation and selection

An essential consideration prior to running any investigation is the validation of the experimental stimuli to be used. Indeed, in order for interpretation biases to be reliably explored, the stimuli used must be truly ambiguous in nature wherein multiple, alternative interpretations are possible. Although single homographs or homophones are commonly used, unless the dominance of alternative meanings is approximately the same, certain meanings are likely to be more apparent than others to all participants. For example, “pain” has a greater set size (i.e., more strong associations) than “pane” (Nelson et al., 2004), and is therefore unlikely to be suitable for use as an ambiguous homophone. Upon hearing this homophone the majority of participants are likely to interpret this as “pain,” thus reducing the likelihood of between-group differences emerging regardless of whether or not they exist in the population (e.g., chronic pain vs. healthy controls). Stimuli validation is equally important for visual scenes and written scenarios, which although may appear ambiguous could nevertheless favor one interpretation over others. Ambiguity may be assessed in a number of ways, but should involve the recruitment of an independent sample of participants providing stimuli ratings. For example, participants may be asked to directly rate the stimuli ambiguity on a visual analog scale, or rate their level of agreement of a list of alternative interpretations presented. The results of these ratings can be used to retain only truly ambiguous stimuli for subsequent research. In addition, stimuli properties of valence and arousal may also be collected (Bradley and Lang, 1994), as it may be presumed that ambiguous stimuli will feature levels of valence and arousal intermediate between neutral and threatening stimuli (indeed, asking participants to provide ratings of stimuli valence is one method of indirectly exploring ambiguity resolution).

Further to ambiguity, valence and arousal, we recommend additional stimuli properties are measured and controlled for, especially if the researcher is comparing biases across multiple stimuli conditions. Considering single words, a strong case can be made for controlling frequency of use and word length, as variations can influence participant responses (e.g., Diependaele et al., 2012; Mueller and Thanasuan, 2014). For ambiguous scenarios, readability and sentence length can also be controlled for, although this is not always reported in the literature. Considering ambiguous visual scenes, it has been argued that the low-level features (e.g., complexity, luminance, color saturation) should be assessed and controlled for (Nummenmaa et al., 2006; Schoth et al., 2015), which may influence cognitive processes of attention and interpretation. Ambiguous video clips have been used much less frequently, although may be standardized by use of the same model/s, with the same clothes, depicted in the same environment. The same is true for confederates of the researcher performing ambiguous behaviors, although variations in confederate performance may still be observed despite careful instructions and training. Overall, we recommend researchers fully report stimuli properties along with any steps taken in stimuli matching and validation. Doing so allows for more reliable comparison of results between studies, as any potential differences in study outcomes may be explained by differences in stimulus properties and matching procedures.

A further issue related to stimuli selection is the specificity of bias to be explored, as a noted limitation of certain studies has been a lack of disorder-related content within the paradigm used (Rohrbacher and Reinecke, 2014). For example, Eley and colleagues explored associations between interpretation biases for a broad range of ambiguous homophones (i.e., mug, leaves, bit, fine, thick and patient) and anxiety and depressive symptoms in children (Eley et al., 2008). The lack of disorder-specific stimuli limits the conclusions which can be drawn from such stimuli however, including whether children with heightened anxiety and depression differ in the specificity of their biased interpretations. In another example, some studies exploring interpretation biases in chronic pain have made use of general illness-related homophones/homographs, as opposed to more specific pain-related stimuli (e.g., Pincus et al., 1994, 1996; for a detailed review see Schoth and Liossi, 2016). Once again, in order to make claims of specific forms of cognitive bias, specific stimuli are required. In combination with the need to report and match stimulus properties, the desire to explore specific forms of cognitive bias may exclude certain paradigms from use due to lack of appropriate stimuli.

### Direct and indirect measures of interpretation biases

Paradigms can provide direct and/or indirect measures of bias. The outcome of direct measures are based on the participant's response (e.g., verbally stating or providing in writing their interpretation), while the outcome of indirect measures are taken from a measure of performance behavior (e.g., response latencies; De Houwer and Moors, 2010)^1^. Resolution of ambiguity typically involves the activation of various potential explanations followed by the selection of a single explanation deemed to be most likely (Wisco, 2009). A limitation of certain paradigms providing a direct measure of bias is that they are unable to distinguish between these different subcomponents of ambiguity resolution. For example, even though paradigms such as the homographic response task and ambiguous scenarios test may instruct participants to write the first response they think of, participants may instead reflect on the possible answers and not provide their initial response. Related to this, a further limitation with direct measures of interpretation bias is the possibility of demand characteristics such as social desirably, or participants' awareness of study aims, influencing the responses provided. This may be less likely to occur with indirect measures of bias as long as the study aims and hypotheses are not revealed to participants. Despite potential limitations however, direct measures may still be used to explore biased interpretations, although we recommend steps are taken to reduce unwanted influences on participant responses. For example, studies may be described neutrally as “language” experiments rather than using more revealing descriptions, and critical trials can be embedded among dummy trials to add weight to this guise (e.g., Edwards and Pearce, 1994). It should also be remembered that there are numerous advantages to direct measures. For example, due to the simplicity of paradigms such as the homographic response task, including relatively straightforward instructions and potentially short duration, these may be more favorable for certain populations such as younger children with shorter attention spans.

Relatively little research has compared the results of direct and indirect measures of biased interpretations in the same sample, although one study exploring biases in high pain-catastrophizers revealed a different pattern of results between direct and indirect measures (Khatibi et al., 2014). This study from Khatibi and colleagues, which used an incidental learning task (indirect measure) and an emotion recognition task (direct measure), is good example of how the same stimuli (in this instance morphed facial expressions) may be used across the two measures. A general recommendation for future research is therefore inclusion of both measures of bias into the experimental design where possible. Such research will help address the extent to which direct measures are more influenced by demand characteristics compared to indirect measures, and whether this is more likely for certain populations than others (e.g., children compared to adults). In order to be useful however, direct measures should be presented following indirect measures. It should also be noted that, although uncommon, different measures of interpretation bias may be recorded concurrently during the same paradigm. For example, several studies using the lexical decision task have recorded behavioral responses and event-related potentials (e.g., Moser et al., 2008, 2012).

### Further recommendations

Considering the strengths and limitations noted for each paradigm (Table 1), we offer further recommendations. For paradigms using single ambiguous words, we recommend researchers stop using paper-based versions where possible. Although relatively simple to administer, it is difficult to control stimuli presentation times which as noted may influence patterns of interpretation. Computerized versions accurately controlling presentation times are preferable, and indeed all of the paradigms identified may be administered via computer. The use of laptop computers also allows for testing in participants homes or schools. For paradigms using ambiguous images, we recommend against the use of the emotion recognition task with unaltered emotional expressions only, which are easy to decode and likely to produce ceiling effects. Use of morphed facial expressions allows for the development of stimuli of different intensities, and will likely be more suitable for detecting differences between participant groups should they exist. A greater number of paradigms have been developed using ambiguous scenarios, some of which differ by relatively small variations in task design and/or requirements only. For example, nine versions of the ambiguous scenarios test were identified, including a modified version in the ambiguous situations task, although all may use the exact same stimuli. Where possible, we recommend researchers using this specific paradigm collect multiple forms of participant response (Stopa and Clark, 2000). This could include asking participants to first imagine the scenario and rate their level of concern or the pleasantness of the scenario, which would provide an initial response less influenced by demand characteristics. Subsequently, participants could then provide the first explanation thought of, or rate their level of agreement of possible interpretations. Using multiple response measures with the same stimuli has the advantage of explicitly testing whether one measure results in a different pattern of responses compared to other measures. This same process may also be used for other types of stimuli, including ambiguous scenes and video clips. Further to issues related to stimuli selection and paradigm choice, it is essential researchers also measure and control potentially important individual difference variables such as years of education and verbal fluency, which may influence participant responses. Finally, the majority of paradigms have been developed with the aim of exploring negative/threatening interpretation biases, although positive interpretation biases also exist and have been associated with improved well-being (e.g., Kleim et al., 2014). Although, few studies have focused explicitly on positive interpretation biases, almost all paradigms identified in the present review could be modified for their exploration.

## Conclusion

The results of this systematic review reveal a broad range of experimental paradigms available for exploring biased interpretation of ambiguous information with threatening/emotional and neutral meanings. A great deal of research has successfully explored such biases in a range of populations, most notably patients with anxiety and depressive disorders, but also in other populations such as patients with chronic pain, anorexia nervosa, and alcohol dependency. One of the challenges for researchers new to this field is the selection of an appropriate paradigm, and it is hoped that this review will help assist in this selection process.

## Author contributions

All authors listed, have made substantial, direct and intellectual contribution to the work, and approved it for publication.

## Funding

This review was funded by an ERSC (http://www.esrc.ac.uk) Ph.D. studentship awarded to the first author (Grant number: ES/I904026/1). The funders had no role in study design, data collection and analysis, decision to publish, or preparation of the manuscript.

### Conflict of interest statement

The authors declare that the research was conducted in the absence of any commercial or financial relationships that could be construed as a potential conflict of interest.

## Footnotes

^1^A very similar way of conceptualizing paradigms is into those providing offline and online measures of bias, although these definitions do vary to those of direct and indirect measures and so are worth noting here. Offline measures are defined as those which allow for reflection on the ambiguous stimulus presented, while online measures are defined as those which record the participant's initial interpretation (Hirsch et al., 2016).
